# Complete Genome Sequence of *Rhodovulum sulfidophilum* DSM 2351, an Extracellular Nucleic Acid-Producing Bacterium

**DOI:** 10.1128/genomeA.00388-15

**Published:** 2015-04-30

**Authors:** Nobuyoshi Nagao, Yuu Hirose, Naomi Misawa, Yoshiyuki Ohtsubo, So Umekage, Yo Kikuchi

**Affiliations:** a Department of Environmental and Life Sciences, Toyohashi University of Technology, Tempaku, Toyohashi, Aichi, Japan; b Electronics Inspired-Interdisciplinary Research Institute (EIIRIS), Toyohashi University of Technology, Tempaku, Toyohashi, Aichi, Japan; c Department of Environmental Life Sciences, Graduate School of Life Sciences, Tohoku University, Sendai, Miyagi, Japan; d Graduate School of Advanced Science and Engineering, Waseda University, Shinjuku-ku, Tokyo, Japan

## Abstract

*Rhodovulum sulfidophilum* DSM 2351 is the nonsulfur photosynthetic bacterium that efficiently releases nucleic acids into the extracellular milieu, which leads to flocculation. In this study, we determined the complete genome sequence of *R. sulfidophilum* DSM 2351, which will provide new insights into the mechanism of its unique nucleic acid release.

## GENOME ANNOUNCEMENT

*Rhodovulum sulfidophilum* is a nonsulfur photosynthetic bacterium, which can grow under both aerobic-dark and anaerobic-light conditions (1, 2). This bacterium releases intracellular macromolecules, such as DNA and RNA, into the extracellular milieu, leading to the formation of cell aggregates, a process known as flocculation (3). We found that *R. sulfidophilum* DSM 2351 shows much higher extracellular nucleic acid production and flocculation rates than those of *R. sulfidophilum* DSM 1374 (our unpublished data), although the two strains are closely related (99.6% similarity in 16S rRNA sequence) (2). To date, the genome sequence of *R. sulfidophilum* is available only for the DSM 1374 strain (4). In this study, we determined the genome sequence of the DSM 2351 strain to investigate the mechanism of its high extracellular nucleic acid production.

Sequencing was performed using the 454 GS FLX+ (Roche) and MiSeq (Illumina) systems. For 454 sequencing, fragment and paired-end libraries were constructed with a GS FLX+ library preparation kit (Roche) and GS FLX paired-end kit (Roche), respectively. We obtained 259,087 fragment reads and 287,202, 308,517, and 318,239 paired-end reads with insert sizes of 8 kbp, 20 kbp, and 30 kbp, respectively. For MiSeq sequencing, a paired-end library and mate-pair libraries were constructed with the TruSeq DNA PCR-free sample preparation kit (Illumina) and the Nextera mate-pair sample preparation kit (Illumina), respectively. We obtained 2,201,763 paired-end reads, with an insert size of 800 bp. The mate-pair libraries yielded 2,096,763 and 1,962,031 reads, with insert sizes of 8 kbp and 12 kbp, respectively. All reads (total, 1.4 Gbp; coverage, 300×) were assembled using Newbler version 2.9 (Roche), yielding 31 scaffolds consisting of 145 contigs. Sequence gaps between the scaffolds and contigs were determined *in silico* using GenoFinisher and AceFileViewer (5), followed by PCR and Sanger sequencing. The finished sequence was validated by FinishChecker, an accessory tool of GenoFinisher.

We succeeded to determine the complete genome sequence of the DSM 2351 strain, which consists of one circular chromosome (4,454,432 bp) and three circular plasmids: plasmid 1 (111,306 bp), plasmid 2 (106,137 bp), and plasmid 3 (60,897 bp). Notably, this is the first report of a complete genome sequence of a member of the genus *Rhodovulum*. Gene prediction and annotation were performed using Microbial Genome Annotation Pipeline (6). The chromosome contains three copies of rRNA operons, 50 tRNA genes, and 4,295 protein-coding genes. Plasmids 1, 2, and 3 carry 89, 88, and 48 protein-coding genes, respectively. The chromosome size of the DSM 2351 strain is approximately 300 kbp larger than that of the DSM 1374 strain (4). Plasmids 1 and 2 are conserved in both strains, but plasmid 3 is found only in the DSM 2351 strain. These differences may result in the different productivities of extracellular nucleic acids between the two strains. Recently, we have developed a new method for the extracellular production of artificial recombinant RNAs using the DSM 1374 strain (7–10). The genome data of the DSM 2351 strain provide new insights into the mechanism of extracellular nucleic acid production of *R. sulfidophilum*, which will contribute to the improvement of our production method.

### Nucleotide sequence accession numbers. 

The complete genome sequence of *R. sulfidophilum* DSM 2351 has been deposited in DDBJ/ENA/GenBank under the accession numbers AP014800 (chromosome), AP014801 (plasmid 1), AP014802 (plasmid 2), and AP014803 (plasmid 3).

## References

[1] Hansen TA, Veldkamp H 1973 *Rhodopseudomonas sulfidophila*, nov. spec., a new species of the purple nonsulfur bacteria. Arch Mikrobiol 92:45–58. doi:10.1007/BF00409510. 4725822

[2] Hiraishi A, Ueda Y 1994 Intrageneric structure of the genus *Rhodobacter*: transfer of *Rhodobacter sulfidophilus* and related Marine species to the genus *Rhodovulum* gen. nov. Int J Syst Bacteriol 44:15–23. doi:10.1099/00207713-44-1-15.

[3] Suzuki H, Daimon M, Awano T, Umekage S, Tanaka T, Kikuchi Y 2009 Characterization of extracellular DNA production and flocculation of the marine photosynthetic bacterium *Rhodovulum sulfidophilum*. Appl Microbiol Biotechnol 84:349–356. doi:10.1007/s00253-009-2031-7. 19452150

[4] Masuda S, Hori K, Maruyama F, Ren S, Sugimoto S, Yamamoto N, Mori H, Yamada T, Sato S, Tabata S, Ohta H, Kurokawa K 2013 Whole-genome sequence of the purple photosynthetic bacterium *Rhodovulum sulfidophilum* strain W4. Genome Announc 1(4):e00577-13. doi:10.1128/genomeA.00577-13. 23929476 PMC3738892

[5] Ohtsubo Y, Maruyama F, Mitsui H, Nagata Y, Tsuda M 2012 Complete genome sequence of *Acidovorax* sp. strain kks102, a polychlorinated-biphenyl degrader. J Bacteriol 194:6970–6971. doi:10.1128/JB.01848-12. 23209225 PMC3510582

[6] Sugawara H, Ohyama A, Mori H, Kurokawa K 2009 Microbial genome annotation pipeline (MiGAP) for diverse users. Abstr 20th Int Conf Genome Informatics (GIW2009), 14 to 16 December 2009, Yokohama, Japan.

[7] Suzuki H, Ando T, Umekage S, Tanaka T, Kikuchi Y 2010 Extracellular production of an RNA aptamer by ribonuclease-free marine bacteria harboring engineered plasmids: a proposal for industrial RNA drug production. Appl Environ Microbiol 76:786–793. doi:10.1128/AEM.01971-09. 19966026 PMC2813014

[8] Suzuki H, Umekage S, Tanaka T, Kikuchi Y 2011 Artificial RNA aptamer production by the marine bacterium *Rhodovulum sulfidophilum*: improvement of the aptamer yield using a mutated transcriptional promoter. J Biosci Bioeng 112:458–461. doi:10.1016/j.jbiosc.2011.07.025. 21903467

[9] Kikuchi Y 2010 Extracellular nucleic acids of the marine phototrophic bacterium *Rhodovulum sulfidophilum* and related bacteria: physiology and biotechnology, p 55–67. *In* Kikuchi Y, Rykova EY (ed), Extracellular nucleic acids. Springer-Verlag, Heidelberg, Germany.

[10] Nagao N, Suzuki H, Numano R, Umekage S, Kikuchi Y 2014 Short hairpin RNAs of designed sequences can be extracellularly produced by the marine bacterium *Rhodovulum sulfidophilum*. J Gen Appl Microbiol 60:222–226. doi:10.2323/jgam.60.222. 25742972

